# Silicon inhibits gummosis by promoting polyamine synthesis and repressing ethylene biosynthesis in peach

**DOI:** 10.3389/fpls.2022.986688

**Published:** 2022-11-28

**Authors:** Huaifeng Gao, Xuelian Wu, Xiaoqing Yang, Maoxiang Sun, Jiahui Liang, Yuansong Xiao, Futian Peng

**Affiliations:** State Key Laboratory of Crop Biology, College of Horticulture Science and Engineering, Shandong Agricultural University, Tai-An, China

**Keywords:** silicon, ethylene, gummosis, polyamine, peach

## Abstract

Silicon is a beneficial element for plant growth, as well as for improving plant resistance to multiple biotic and abiotic stresses. Gummosis is a common harmful disease in peach and is induced by many factors. However, the effect of silicon on gummosis of peach has not been determined yet. In this study, we reported that application of silicon significantly reduced gummosis by regulating biosynthesis of ethylene and polyamines in peach. Ethylene promoted the development of gummosis by inducing the expression of genes encoding cell wall degrading enzymes. While application of different types of polyamines, including spermidine and spermine, dramatically inhibited the occurrence of gummosis. Moreover, polyamines inhibited the ethylene biosynthesis by down-regulating expression of ethylene biosynthetic gene *PpACS1* (1-aminocyclopropane -1-carboxylic acid synthase), as well as the enzymatic activity of ACS. We further found that application of silicon significantly restricted the development of gummosis in peach. Exogenous silicon dramatically inhibited expression of *PpACS1* and the enzymatic activity of its product to reduce ethylene biosynthesis. Simultaneously, the activity of S-adenosylmethionine decarboxylase, a key enzyme in ployamines biosynthesis, was increased by 9.85% under silicon treatment, resulting in elevated accumulation of polyamines. Thus, our data proved that application of silicon restricted gummosis development by activating ployamines biosynthesis and inhibiting ethylene synthesis in peach.

## Introduction

Gummosis is a harmful disease that can be induced by a variety of factors. Gummosis is a broad defence response to abiotic and biotic stresses, accompanied by a series of complex physiological and biochemical reactions (Li et al., 2015; Zhang et al., 2022). The disease is common in cultivation of peach, cherry, plum, and rose (Saniewski et al., 1998; Saniewski et al., 2001; Adesemoye et al., 2014), and has been reported in many countries, including China, the United States, Japan, and so on (Wang et al., 2011; Adesemoye et al., 2014; Ezra et al., 2017). Excessive gum formation seriously weakens tree vigour, destroys the branches and fruits, resulting in decreased yield and quality of fruit. Gummosis involves a process of accumulation and penetration of polysaccharides, and the formation of gum is due to the degradation of cell wall. The gum comes from polysaccharides in the cell wall and is produced in the cambium (Simas-Tosin et al., 2010; Vincent et al., 2010; Li et al., 2014a). The resulting changes in cell wall structure are mainly due to the degradation of pectin and cellulose and changes in other cell wall components. Gum is formed through the hydrolysis of cell wall substances, which is closely related to the activities of hydrolases such as polygalacturonase (PG), pectin-methylesterase (PME) and β-galactosidese (β-Gal) (Vorwerk et al., 2004).

There are many factors that cause gummosis, which mainly include pathogen infection, stress, freezing damage, pest damage, and mechanical wounds (Li et al., 2014a). In addition, plant hormones such as ethylene and jasmonic acid are important factors which exacerbate cell senescence and lysis and accelerate gum production (Miyamoto et al., 2010; Li et al., 2014b; Li et al., 2015). Studies have shown that ethylene promotes the production of gum in plant under stress conditions. Exogenous ethephon induces gum formation in cherry (Miyamoto et al., 2019), hyacinth (Miyamoto et al., 2015), and peach (Li et al., 2015). In addition, concurrent application of ethephon and methyl-jasmonate enhances gummosis in peach (Li et al., 2015), cherry (Miyamoto et al., 2019) and *Prunus mume* (Carolina and Kusumoto, 2020). Ethylene is widely present in various organs of plants, and its synthesis is a methionine cycle with two key enzymes, ACC (1-aminocyclopropane-1-carboxylic acid) synthase (ACS) and ACC oxidase (ACO). ACS catalyses the synthesis of ACC from S-adenosylmethionine (SAM), and ACO catalyses the synthesis of ethylene from ACC. ACS and ACO are encoded by a multigene family, and transcription is regulated by multiple factors (Ye et al., 2017; Yuan et al., 2019).

Polyamines are essential aliphatic compounds that have multiple functions in plant growth and development and as wll as in cell homeostasis (Bregoli et al., 2006; Leonardo et al., 2010). They are involved in protective stress responses, and the level of polyamine biosynthesis is significantly increased under stress conditions (Franchin et al., 2007). SAM is catalytically decarboxylated by SAM decarboxylase (SAMDC). In addition to serving as a methyl donor, decarboxylated SAM is also a common precursor for the synthesis of polyamines and ethylene, so SAMDC is considered being a regulator of two synthetic pathways. Silicon was shown to enhance the expression of the SAMDC gene, which encodes a key enzyme in the biosynthesis of polyamines, thereby promoting polyamine synthesis and inhibiting ethylene synthesis (Yin et al., 2016). Ethylene and polyamines are synthesized in a competitive relationship, and polyamines share a common substrate, SAM, with ethylene and produce a common product, 5’-methylthioadenosine (Pandey et al., 2000). They have opposite functions, ethylene as a pro-senescence, pro-ripening regulator while polyamines as pro-growth, anti-senescence regulators, have drawn more attention to them (Harpaz-Saad et al., 2012).

Silicon is beneficial to plant growth, especially under stress conditions (Ma and Yamaji, 2006; Safoora et al., 2018). It has been shown to alleviate various abiotic stresses, including heavy metal, salinity, drought, etc. (Gong and Chen, 2012; Bharwana et al., 2013) and biotic stresses, including as pests and diseases (Rodrigues et al., 2004; Liang et al., 2010; Shirani and Zheng, 2018). Moreover, application of silicon improves plant disease resistance. Studies have found that silicon improved plant resistance to rice blast, bacterial blight, and cucumber and melon powdery mildew (Guo et al., 2005; Yu et al., 2009; Wen, 2016). Silicon also activates the plant inducible defence system through physiological and biochemical defence mechanisms to enhance resistance to diseases (Ning and Liang, 2014). Silicon was reported to increase the levels of polyamines, such as free and bound putrescine (Put), by promoting the polyamines biosynthetic genes in sorghum tissues under salt stress (Yin et al., 2019). The study also found that silicon induced the expression of genes related to plant defence and interacted with disease resistance signalling molecules (such as salicylic acid, jasmonic acid, and ethylene) in signal transduction (Chain et al., 2010). Specifically, silicon induces the expression of genes that are involved in cell wall structural modification, allergic response, synthesis of hormones and antibacterial complexes, and pathogenesis-related proteins (Bélanger et al., 2003; Fauteux et al., 2006).

Silicon is beneficial for improving plant disease resistance, but there is no research on whether it can enhance peach gummosis resistance. And it is not clear whether silicon can inhibit the occurrence of peach gummosis by regulating the synthesis of ethylene and polyamines. In this study, we found that silicon inhibited peach gummosis. To better understand the mechanism of gummosis inhibited by silicon in peach, we investigated the factors that influence gummosis caused by ethylene and analyzed the expression patterns of related genes during progression of gummosis inhibited by silicon in peach. We found that application of silicon restricted gummosis development by activating ployamines biosynthesis and inhibiting ethylene synthesis in peach.

## Materials and methods

### Plant materials and treatments

Throughout the experiments, shoots were collected from 3-year-old *Prunus persica* (L.) ‘Spring snow’ plants. The plants were grafted onto wild peach rootstocks and grown in the experimental field of Shandong Agricultural University in Taian. The shoots were cut into 12.0-cm-long segments and surface-sterilized with 75% alcohol for 10 s, followed by rinsing three times with sterile water. The shoot segments were wounded at intervals of 0.5 cm on the midpoint using a sterilized needle. Treatments with silicon, ethephon and various reagents were performed as follows: Na_2_SiO_3_ (0.6 mmol/L), ethephon (1.0%, w/w), Put(0.1 mmol/L), Spermidine (Spd) (0.1 mmol/L), Spermine (Spm) (0.1 mmol/L), D-Arginine (D-Arg) (10.0 mg/L), ACC (50 mg/L), Aviglycine (AVG) (0.5 mmol/L) and Methylglyoxal bis-guanyl hydrazine (MGBG) (0.5 mmol/L) were applied individually or together on selected peach shoots; as a control, water was sprayed on the shoots. The shoot segments were separately placed in an upright position in 2000 mL plastic bottles that contained 200 mL of sterilized water, and the bottles were covered with transparent plastic film to maintain humidity. The water in the bottles was refreshed daily, and the isolated shoots were cultivated in an incubator with a humidity of > 70% at 35°C and a photoperiod of 12 hours of light (20,000 lux). Each treatment was made up of 30 peach segments, and six independent segments were obtained in the same way from the treated and control groups for sample collection. The treatment was continued for 5 days. Gum formation was described according to Saniewski et al. (1998). Samples were taken at 0 h, 6 h, 1 day, 2 days and 4 days after treatment, and the tissue within 0.5-1.0 cm from the wound point was collected from the segments. The samples were promptly frozen in liquid nitrogen and then kept at a temperature of -80°C for later use.

### Preparation of peach shoots tissues for RNA-Seq and analyze

The test material was 3-year-old *Prunus persica* (L.) ‘Spring snow’ plants. The current year’s shoots of uniform growth were selected, and samples were taken on the third day after treatment with ethephon (1.0%, w/w). The control was treated with clean water.

The experimental process of sequencing was carried out by HuaDa Gene (http://www.genomics.cn/index). For this work, the Illumina Gene Expression Sample Prep Kit and Solexa Sequencing Chip (flow cell) were used, and the main instruments were an Illumina Cluster Station and Illumina HiSeq TM 2000 System.

### Measurement of ethylene production

Six peach shoots subjected to different treatments were selected and cultivated in a closed beaker for 3 h. One millilitre of gas from the beaker’s headspace was sampled after incubation. A gas chromatograph (GC-2014C, Shimadzu, Japan) equipped with a GDX-502 column and a flame ionization detector was used to measure ETH production (FID). The injection temperature was 120°C, and the column temperature was 70°C. N_2_was used as the carrier gas at a rate of 40 mL min^-1^.

### ACC measurement

Two millilitres of 85% ethanol was used to extract 0.5 g (fresh weight) of plant material. Following drying of the extracted supernatant, 1.0 mL of chloroform and 1.0 mL of distilled water were added separately. Then, HgCl_2_ and NaOCl/saturated NaOH (2:1) were added to the aqueous phase. After incubation and intermittent agitation, 1 mL of the air in the headspace was extracted with a gas syringe and injected into a gas chromatograph (GC-2014C, Shimadzu, Japan). The aqueous phase was hydrolysed in 6.0 M HCl at 100°C for 3 h to quantify malonyl-ACC (MACC, conjugated ACC).

### Polyamine quantification

High-performance liquid chromatography was used to examine the polyamine content (HPLC: LC-1290, Agilent, America). Plant materials (1.0 g) were pulverized and homogenized in 5.0 mL of 5.0% HClO_4_ and extracted overnight at room temperature on a shaker. The collected supernatant was used to determine the amount of free polyamines after centrifugation. To measure the bound polyamines (conjugated polyamines and macromolecules), the residue of the plant extract was washed with 5.0% HClO_4_ and then hydrolysed in 6.0 M HCl at 110°C for 15 h. The filtered hydrolysate was evaporated to dryness, and the residue was dissolved in 5% HClO_4_ for quantification of the bound polyamines.

### Enzyme activity assay

An enzyme-linked immunosorbent assay kit (Meibiao Biology, Jiangsu Meibiao Biology Technology Company Limited) was used to detect the enzymatic activity of ACS, ACO, PG, PME, β-Gal and SAMDC. Plant samples (0.5 g) were ground in liquid nitrogen, 5.0 mL of PBS (pH 7.4) was added, and the samples were fully homogenized by a homogenizer. The samples were centrifuged at 3000 rpm for approximately 20 minutes, and the supernatant was collected. Samples were added according to the instructions and detected with a microplate reader, and then the enzyme activity was calculated according to the standard curve.

### RNA extraction and quantitative PCR

According to the manufacturer’s instructions, an RNAprep Pure Plant Kit (Tiangen, Beijing, China) was used to isolate total RNA from 0.5 g of sample. The PrimeScriptTM RT Kit (Takara, Japan) was used to generate first-strand cDNA according to the manufacturer’s instructions. On a QuantStudio^®^ 3 real-time PCR instrument (Thermo, USA), we performed three biological and three technical duplicate RT-qPCRs using SYBR^®^ Premix Ex TaqTM (Takara, Japan), with *PpActin* as the reference gene, according to the manufacturer’s recommendations for all reagents. The 2^−ΔΔCT^ approach was used to calculate the relative expression levels. (Primers are shown in **Table 1**)

**Table 1:** Primers used in this study.

Primer	Primer sequence (5′→3′)
*ppa007271m*-F	GCATCCCTAAACAGCCAAAT
*ppa007271m*-R	GCTCCCAATACTGATTCCATG
*ppa003578m*-F	CCAGACAATCATCACAGGAAACA
*ppa003578m*-R	GCGTGTATAAGGTGTCTTGGTAGC
ppa001382m-F	TGGTAGTGTTTGAAGAATGGGGT
ppa001382m-R	ATGGGCTTTTGGTCTGTTGGTT
*PpACS1*-F	GGCAAGGTTCCTGGAGACAA
*PpACS1*-R	CACAATCACACGCCAAAGCA
*PpACS2*-F	TGCACAGCAGCAGGAGTAAA
*PpACS2*-R	CCAGGATCAGCCAAGCAGAA
*PpACO*-F	GCAACTACCCTCCTTGTCCC
*PpACO*-R	TGGCCATCTTTGAGGAGCTG
*PpSAMDC*-F	AGGGGATACTGTTGCGGAGA
*PpSAMDC*-R	CGCTCCAGCAACCTTTCAGA
*Ppactin* -F	TGCATTGTGTATGTGTTCATCTACA
*Ppactin* -R	CTTCACCATTCCAGTTCCATTGTC

### Statistical analysis

The biological and biochemical data are presented as the means ± standard errors (SEs). The significance of the differences between samples was assessed by Duncan’s multiple range tests using IBM SPSS Statistics version 20.0.

## Results

### Application of ethephon promoted the development of gummosis in peach shoots

We found that treatment with needle wounding induced the gum production on peach shoots, while no gum was found in control treatment (**Figure S1A**). However, co-treatment with wounding and 1% ethephon significantly promoted the production of gum compared with that in wounding treatment (**Figures S1B, C**). These data suggested that ethylene was a promoting factor for gummosis in peach.

### Transcriptomic analysis of genes expressed during ethephon-induced gummosis in peach using DGE profiling

To determine the mechanism of how ethylene facilitated gummosis on peach shoots, we constructed a digital gene expression profile library from ethephon-treated and untreated peach shoots. Data analysis showed that many genes were induced or inhibited by more than 2-fold in the bark tissue of peach shoots 3 d after ethephon treatment (**Figure S2A**). There were 2.13% of tags in ethephon-treated samples having signatures upregulated by more than 5-fold, and 2.74% in CK when the variation in signatures of differentially expressed genes between ethephon and CK was within 6-fold (**Figure S2B**). According to the screening criteria for differential gene expression, 1362 genes were upregulated and 3005 genes were downregulated under the ethephon treatment (**Figure S2C**). Among the differentially expressed genes, three kinds of key enzymes that were related to cell wall degradation were identified. Among them, two polygalacturonase genes (*ppa007271m, ppa008474m*) and three pectinesterase genes (*ppa003578m, ppa003852m, ppa003639m*) were significantly induced by ethephon. Especially for *ppa003578m* (pectinesterase) and *ppa007271m* (polygalacturonase), which were upregulated by 12.176- and 6.620-fold, respectively (**Table 2**).

**Table 2:** DEGs involved in cell wall degradation.

BiologicalFunction	Gene ID	Gene description	log2FoldChangeEthephon vs CK
Cx-cellulase	ppa005285m	Cellulase	0.196
	ppa002911m	Cellulase1	-1.275
Polygalacturonase	ppa007271m	Polygalacturonase	6.620
	ppa008474m	Polygalacturonase	2.471
	ppa003003m	Polygalacturonase-1precursor	-6.375
	ppa004793m	Polygalacturonase	-2.151
Pectinesterase	ppa003578m	Pectin methylesterase 3	12.176
	ppa003852m	Pectin methylesterase 1	7.570
	ppa003639m	Pectinesterase-2 precursor	8.139
	ppa003047m	Pectin methylesterase 1	-1.083
	ppa004300m	Pectinesterase inhibitor PPE8B	-0.438
	ppa003697m	Pectinesterase precursor	1.202
	ppa022308m	Pectinesterase-2 precursor	-5.358
	ppa024549m	Pectinesterase inhibitor	-5.358
	ppa003400m	Pectinesterase-3 inhibitor	-2.139
	ppa008873m	Pectinesterase precursor	-0.509
	ppa004300m	Pectinesterase inhibitor PPE8B	-0.438
	ppa003307m	Pectinesterase-3 precursor	-0.274
β-galactosidese	ppa001382m	Beta-galactosidase	-3.425
	ppa001334m	Beta-D-galactosidase	-3.415
	ppa001363m	Beta-D-galactosidase	-2.867
	ppa001479m	Beta-D-galactosidase	-2.680
	ppa007136m	Alpha-galactosidase	-1.710
	ppa001149m	Beta-galactosidase3	-1.200
	ppa001412m	Beta-galactosidase protein 1	-0.024

A value lower than 0 indicates downregulation, and a value higher than 0 indicates upregulation.

### Effects of silicon on gummosis in peach shoots

**Figure 1:**
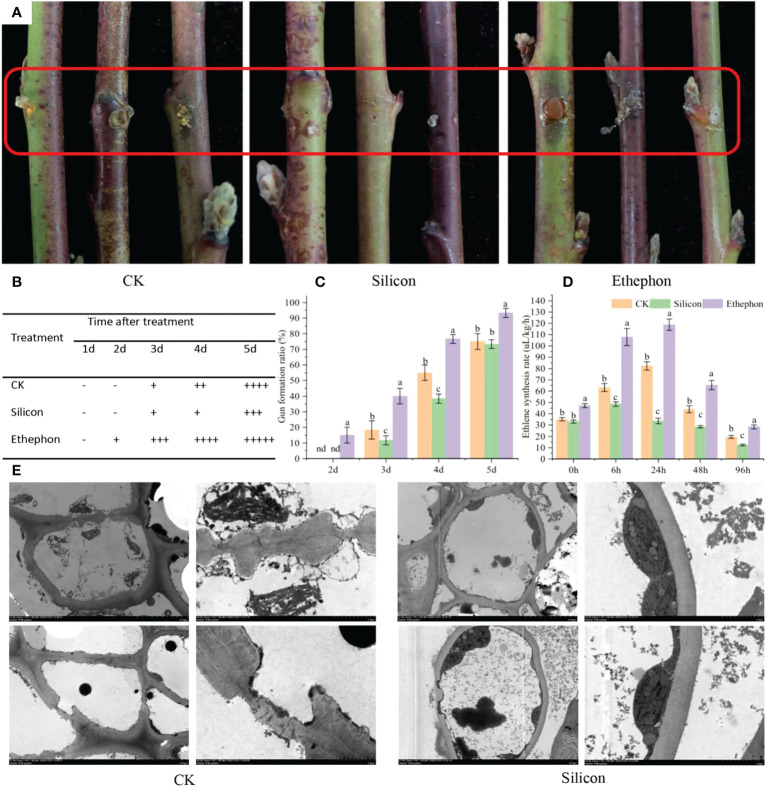
Silicon inhibits gum formation in peach shoots. **(A)** Photograph of gum formation in peach shoots treated with silicon (0.6 mmol/L) or ethephon (1%, w/w) after 5 days. **(B)** Gum formation score of shoots treated with silicon or ethephon after 5 days. The relative levels of gum production were graded based on visual observation, and the scores were as follows: − no gum; + to ++++, increasing degrees (amounts) of gum production (+, trace; ++++, high). **(C)** Gum formation rates of peach shoots treated with silicon or ethephon. **(D)** Ethylene synthesis rate of shoots treated with silicon or ethephon. **(E)** Electron microscopy image of the cell structure after treatment with water and silicon for 5 days (the picture on the right is the marked position of the picture on the left). Data represent the means of three replicates ± SEs, and different letters indicate significant differences (P < 0.05).

As shown in **Figure 1A**, following silicon treatment, the gum on the peach shoots was significantly smaller than that of the control, while a large amount of gum was produced at the wound of the shoots treated with exogenous ethephon. The gum formation ratio under different treatments showed that the gum appeared earlier and accumulated more in ethephon-treated shoots. The gum formation ratio was 24.44% higher than that of control after 5 d. In contrast, silicon inhibited the production of gum on shoots, the gum formation ratio was reduced by 30.30% compared with control after 4 d (**Figures 1B, C**). Importantly, a similar result was observed for ethylene synthesis under different treatments (**Figure 1D**). Silicon inhibited ethylene synthesis in shoots, and the ethylene synthesis rate was reduced by 71.66% compared with ethylene-treated shoots after 24 h of treatment. In electron microscopy analysis, we observed that silicon treatment alleviated the cell damage compared with that of control. Specifically, under silicon treatment, the structure of cell walls and cellular organelles were more intact (**Figure 1E**).

### Effects of silicon on cell wall degradation in peach shoots

**Figure 2:**
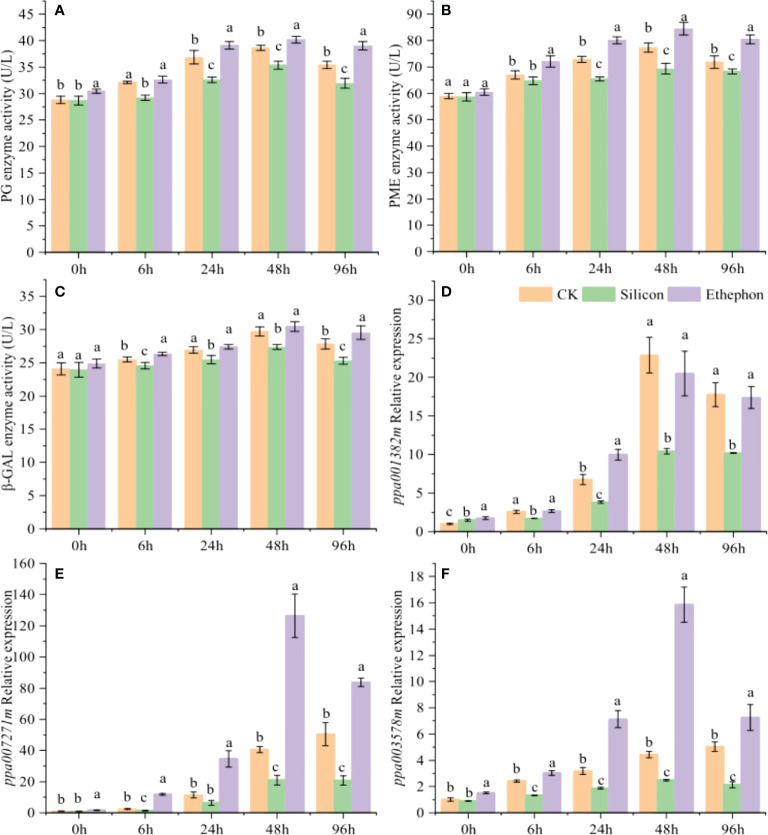
Silicon inhibits cell wall-degrading enzyme activity in peach shoots. Enzyme activity of PG **(A)**, PME **(B)** and β-Gal **(C)** in peach shoots. Relative expression of *ppa007271m* **(D)**, *ppa003578m* **(E)** and *ppa001382m* **(F)** in peach shoots. The peach shoots were treated with water, Na_2_SiO_3_ (0.6 mmol/L) or ethephon (1%, w/w), and samples were taken at different times to determine the enzyme activity and relative gene expression. Bars with different letters indicate significant differences (p < 0.05, Duncan’s multiple range tests).

Transcriptome analysis showed that cell wall-degrading enzymes were closely related to peach gummosis. We thus used an enzyme-linked immunosorbent assay kit to analyse the enzyme activity of PG, PME and β-Gal and screened the cell wall-degrading enzyme synthesis genes *ppa007271m* (PG), *ppa003578m* (PME) and *ppa001382m* (β-Gal), which are significantly regulated by ethylene, for RT–qPCR analysis. The results showed that enzymatic activity of these three enzymes, while silicon founctioned adversely. In particular, the PG and PME enzymatic activity were significantly inhibited by silicon (**Figures 2A–C**). 24 h after treatment, the activity of PG and PME under silicon treatment decreased by 11.61% and 10.16% compared with the control, respectively. The expression of *ppa007271m*, *ppa003578m* and *ppa001382m* in peach shoots were significantly upregulated with the increasing degree of gum formation (**Figures 2D–F**). The expression levels of *ppa007271m* and *ppa003578m* were significantly upregulated by ethephon, while they were all downregulated by silicon, especially after 24 h of treatment.

### Effects of silicon on ethylene synthesis in peach shoots

**Figure 3:**
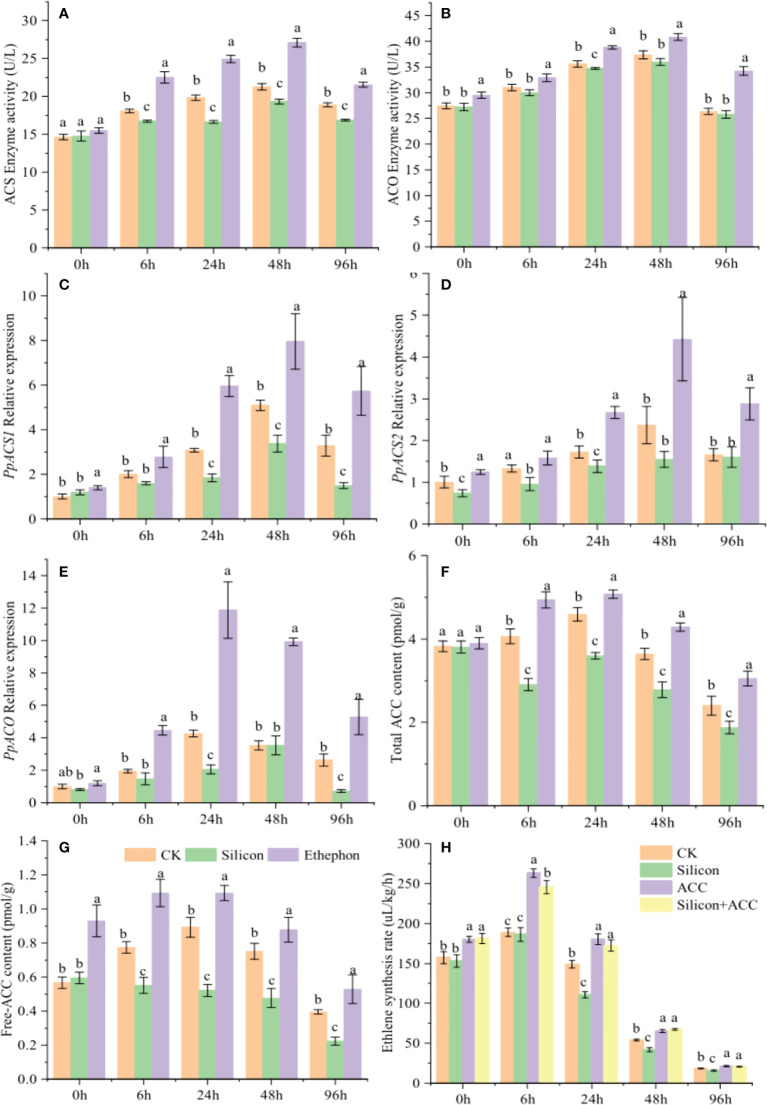
Silicon inhibits ethylene synthesis in peach shoots. Enzyme activity of ACS **(A)** and ACO **(B)** in peach shoots under different treatments. Relative expression of *PpACS1* **(C)**, *PpACS2* **(D)** and *PpACO* **(E)** in peach shoots under different treatments. Total ACC content **(F)** and free ACC content **(G)** of peach shoots treated with different fluids. **(H)** Ethylene synthesis rate of peach shoots treated with exogenous ACC. Bars with different letters indicate significant differences (p < 0.05, Duncan’s multiple range tests).

Given that ethylene synthesis is closely involved in the inhibitory effect of silicon on the gummosis of peach shoots, we next determined the effect of silicon on ethylene biosynthesis. Under silicon treatment, the enzymatic activity of ethylene synthase ACS (**Figure 3A**) and the relative expression levels of *PpACS1* (**Figure 3C**) and *PpACS2* (**Figure 3D**) were all decreased. Compared with the control, they decreased by 7.40%, 39.94% and 19.63% 24 hours after treatment. However, silicon had no effect on the enzymatic activity and gene expression level of ACO (**Figures 3B, E**). Silicon treatment reduced the content of total ACC and free ACC in shoots by 21.72% and 41.67% compared to the control after 24 hours treatment, which was contrary to the results of exogenous ethephon treatment (**Figures 3F, G**). After exogenous ACC treatment, ethylene synthesis was promoted in shoots and that the inhibitory effect of silicon on ethylene synthesis was alleviated by ACC (**Figure 3H**). These results indicated that the effect of silicon on ethylene synthesis in peach shoots was achieved through the ACC pathway.

### Effects of polyamines on ethylene synthesis in peach shoots

**Figure 4:**
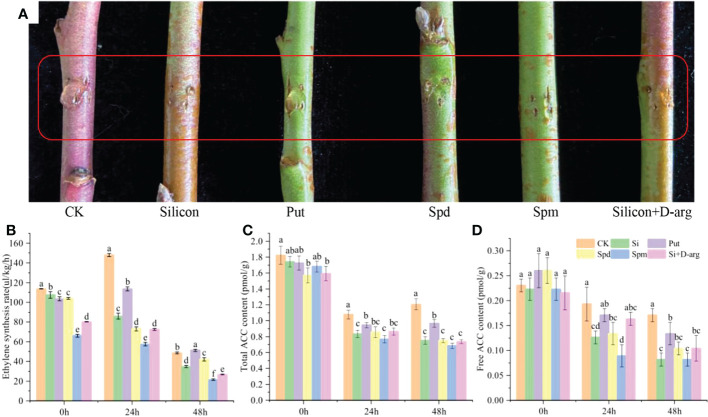
Spd and Spm inhibit ethylene synthesis in peach shoots. **(A)** Photograph of gum formation in peach shoots treated with water, Na_2_SiO_3_ (0.6 mmol/L), Put (0.1 mmol/L), Spd (0.1 mmol/L), Spm (0.1 mmol/L) or Na_2_SiO_3_ +D-Arg (10 mg/L) after 5 days. **(B)** Ethylene synthesis rates of differentially treated peach shoots. Total ACC content **(C)** and free ACC content **(D)** of peach shoots treated with different fluids. Bars with different letters indicate significant differences (p < 0.05, Duncan’s multiple range tests).

In order to investigate the effect of polyamines on ethylene synthesis, Put, Spd, Spm and D-Arg (Putrescine synthesis inhibitor) were used to treat peach shoots. As shown in **Figure 4A**, silicon, Spd and Spm inhibited gum formation, as well as the ethylene biosynthesis on peach shoots. After 24 h treatment, compared with the control, the ethylene synthesis rate was reduced by 41.90%, 50.60% and 61.09%, respectively. However, Put had no inhibitory effect on ethylene synthesis in peach shoots, and accelerated the production of gum on shoots (**Figure 4B**). The total ACC and free ACC levels, as well as the ACS activity, were reduced under treatment of silicon, Spd and Spm after 24 hours-after-treatment (**Figures 4C, D** and **S3A**). The expression of *PpACS1* was downregulated by silicon, Spd and Spm throughout the whole process. The expression of *PpACS1* under treatment of silicon, Spd, and Spm was reduced by 59.10%, 64.76% and 52.43% compared with the control, respectively, after 24 h treatment (**Figure S3B**). While the expression of *PpACS2* was downregulated by silicon, but not Spm and Spd (**Figure S3C**).

### Effect of silicon on the synthesis of polyamines and ethylene

**Figure 5:**
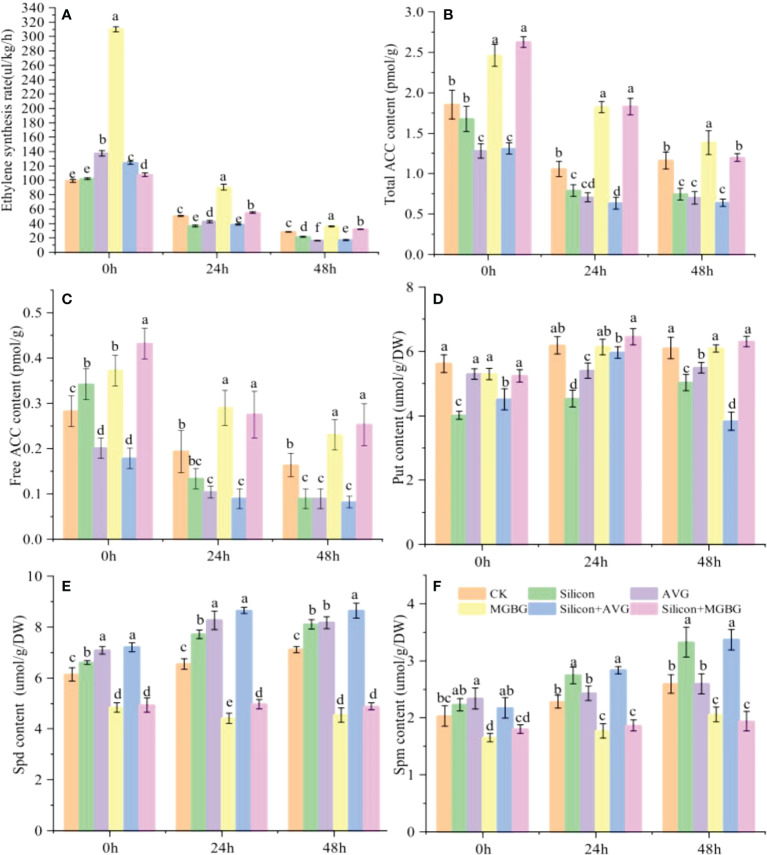
Effect of silicon on ACC and polyamine content. **(A)** Ethylene synthesis rate of differentially treated peach shoots. Total ACC content **(B)** and free ACC content **(C)** of peach shoots treated with different fluids.The Put content **(D)**, Spd content **(E)** and Spd content **(F)** in peach shoots under different treatments.The peach shoots were treated with water, Na_2_SiO_3_ (0.6 mmol/L), AVG (0.5 mmol/L) and MGBG (0.5 mmol/L) individually or in combination, and samples were taken for analysis at 0h, 24h, and 48h after treatment. Bars with different letters indicate significant differences (p < 0.05, Duncan’s multiple range tests).

The ethylene and polyamine synthesis pathways are considered competitive because they share the same precursor, methionine. Under AVG (an ACC synthesis inhibitor) and MGBG (a Spd and Spm synthesis inhibitor) treatment, the synthesis of ethylene and ACC showed opposite results. AVG inhibited the synthesis of ethylene and ACC, while MGBG had the opposite effect. Silicon inhibited ethylene and ACC synthesis as AVG, but the inhibitory effect of silicon was disappeared when peach shoots were co-treated with silicon and MGBG (**Figures 5A–C**). Moreover, silicon treatment increased the content of Spd (17.74%) and Spm (20.27%), but not Put, in peach shoots. AVG increased the content of Spm and Spd, while MGBG functioned adversely. The promoting effect of silicon on the Spm and Spd content was also enhanced by AVG but inhibited by MGBG (**Figures 5D–F**).

In peach shoots, treatment of AVG inhibited the enzymatic activity of ACS, while MGBG inhibited the enzyme activity of SAMDC. Silicon increased the enzyme activity of SAMDC and inhibited ACS enzyme activity. Compared to control, the enzyme activity of ACS was reduced by 11.50%, while that of SAMDC was increased by 9.85% after silicon treatment (**Figures S4A, B**). While the inhibitory effect of silicon on ACS activity was alleviated upon addittion of MGBG. The expression of *PpACS1* and *PpACS2* were downregulated by silicon and AVG, and both of them were upregulated by MGBG. However, the upregulation of gene expression by silicon was weakened by MGBG (**Figures S4C, D**). The expression of the *PpSAMDC* was upregulated 12.68-fold compared to control after silicon treatment, and this effect was enhanced by AVG but attenuated by MGBG (**Figure S4E**). This series of results shows that silicon inhibits the synthesis of ACC by promoting the synthesis of Spm and Spd, thereby inhibiting the production of ethylene.

## Discussion

Gummosis is a process of gum formation, accumulation and exudation that occurs in a variety of plants, especially in the family *Rosaceae* (Miyamoto et al., 2010). Research on plant gummosis found that hormones are important factors affecting its pathogenesis (Miyamoto et al., 2015; Agnieszka et al., 2019). Since all the abiotic and biotic environmental stress factors, such as pathogen infection, stress, pest damage, etc., are considered being mediated by the action of ethylene produced in plant tissues. And exogenous ethylene or ethephon induces gummosis, so ethylene is considered to be the common factor for gummosis (Li et al., 2014b; Li et al., 2015). Our study found that the mechanical damage site of peach shoots was a high-incidence area for gummosis disease, and exogenous ethephon treatment accelerated the rate of gummosis and increased the gum quantity. The process of gummosis is caused by the destruction of the cell wall structure to form gum ducts, and the formation of gum ducts is a process of cell wall degradation (Gedalovich and Fahn, 1985; Agnieszka et al., 2019). By using radioactive labelling of polysaccharides in the cell wall of gum duct epithelial cells of cherry trees, it was found that the gum came from the polysaccharides in the cell wall (Stosser et al., 1978). There are many kinds of cell wall degrading enzymes that are important factors of cell wall degradation. Among which PME, PG and β-GAL are closely related to peach gummosis (Wei et al., 2010). Transcriptome analysis of peach shoots treated with exogenous ethephon showed that ethylene affected the expression of PME, PG and β-GAL-encoding genes, and the expression of *ppa003578m*, *ppa003852m*, *ppa003639m* and *ppa007271m* was significantly upregulated by ethylene. After ethephon treatment, the activity of PME, PG and β-GAL were increased in the gummosis-affected region of peach shoots, indicating that the degradation of the cell wall was aggravated and the formation of gum was accelerated. In addition, ethylene promotes the formation of gum ducts in *Prunus mume* and increases the length and area of gum ducts (Carolina and Kusumoto, 2020). The main reason of peach gummosis is an excessive synthesis of endogenous ethylene.

Silicon is a beneficial element for plant growth and has been shown to improve plant disease resistance (Zeyen, 2002; Sathe et al., 2021).We also found that silicon played an important role in inhibiting peach gummosis, which reduced its incidence. Silicon effectively inhibited the damage to the cells in the gummosis-affected area of the peach shoots and reduced the degradation of the cell wall. On one hand, the strengthening effect of silicon on the cell wall was due to the accumulation of silicon in the shoots. Which promoted the enhancement of cell silicification, enhanced the performance of the cell wall, and formed a physical defence barrier (Cai et al., 2010; Law and Exley, 2011). On the other hand, silicon inhibited ethylene synthase activity and gene expression, preventing massive ethylene synthesis, and reduced the activity of cell wall-degrading enzymes in peach shoots. The regulatory effect of ethylene synthesis by silicon has yet been verified in rice, tobacco and other plants. The inhibitory effect of silicon on rice blast was attributed to silicon inhibiting the expression of ethylene synthesis genes in rice. Rice treated with silicon and subjected to wounding had a lowered ethylene content (Kim et al., 2015; Souri et al., 2020). Silicon can alleviate the salt stress of tobacco suspension cells, but this alleviation must involve the participation of ethylene (Liang et al., 2015).These conclusions also verify our hypothesis. Microarray analysis revealed a stimulatory role of silicon in activating the ethylene signaling pathway in plants infected with pathogens and improving plant immunity (Tripathi et al., 2020). These evidence suggest that silicon inhibits gummosis not only through physical defence but also by inhibiting ethylene synthesis.

Ethylene is synthesized excessively in plants under stress from diseases, insect pests, or mechanical damage (Morgan and Drew, 2010). The immediate precursor of ethylene synthesis is ACC, and its synthesis is regulated by a variety of factors (Kacperska and Maria, 2010). We found that silicon inhibited ethylene synthesis, but exogenous ACC weakens the inhibitory effect of silicon on ethylene synthesis, so silicon reduced ethylene production by inhibiting ACC synthesis. ACS is a key enzyme in ACC synthesis, and the expression of its key synthesis gene *PpACS1* was regulated by silicon. This is consistent with Yin’s results, indicating that silicon mediates the ACC synthesis process (Harpaz-Saad et al., 2012; Yin et al., 2014). Polyamines play a variety of roles in plant growth and development, as well as in cell homeostasis (Bregoli et al., 2006; Pottosin and Shabala, 2014; Nader et al., 2020). Polyamine biosynthesis levels are dramatically enhanced under increased stress and are involved in stress defence responses (Francois et al., 2005; Takahashi et al., 2009; Ashraf et al., 2011). Similarly, we found thatthe polyamine levels increased with silicon application. Silicon increased the synthesis of Spd and Spm by upregulating the expression of *PpSAMDC* and increasing the activity of SAMDC (Ding et al., 2015; Yin et al., 2016). But the enhancement of silicon was blocked by MGBG. SAMDC has a short half-life and is a rate-limiting enzyme in the production of Spd and Spm. Overexpression of SAMDC increases polyamine levels and strengthens resistance (Roy and Wu, 2002), and this is consistent with our results. Polyamines working as stress signalling regulators can induce plant defence responses and enhance resistance (Kuznetsov and Shevyakova, 2010; Singh et al., 2015). Silicon increased the polyamine content, and this may also be a mechanism to inhibit the occurrence of gummosis. Polyamines may be involved in silica precipitation, as long-chain polyamines were discovered to be involved in the mediation of silica precipitation from a silicic acid solution in diatoms (Kröger et al., 2000; Shi et al., 2013). The interaction between silicon and polyamines would have a beneficial effect on the precipitation of silicon on the cell wall and induce plant defence responses.

The enzyme ACS catalyses the conversion of SAM to ACC, which is a critical regulatory step in the biosynthesis of ethylene (Harpaz-Saad et al., 2012). Previous studies had found that polyamines reduced ethylene release by inhibiting ACC synthesis, thereby increasing sorghum’s salt resistance (Yin et al., 2016). After the mutation of the *LeACS6* in tomato, reduced ACS enzyme activity, ethylene synthesis was blocked (Shi et al., 2003). In this experiment, silicon increased the Spd and Spm levels in peach shoots, and RT-qPCR showed that the expression of *PpSAMDC* was upregulated. Furthermore, Spd and Spm inhibited ethylene synthesis by reducing the enzyme activity and gene expressions of ACS, and the levels of total ACC and free ACC were decreased. AVG can promoted the synthesis of polyamines, which had the same effect as silicon. This is consistent with previous research results. However, when silicon and MGBG were used together, the synthesis of Spd and Spm was blocked, and the inhibitory effect of silicon on ACC synthesis was weakened. The results for the enzyme activity and relative gene expressions of ACS and SAMDC supported this conclusion. Ethylene and polyamines have a competitive interaction, implying that silicon inhibits the synthesis of ACC by promoting the synthesis of Spd and Spm, thus inhibiting ethylene synthesis. Decreased accumulation of ACC as well as ethylene can alleviate gummosis.

## Conclusions

**Figure 6:**
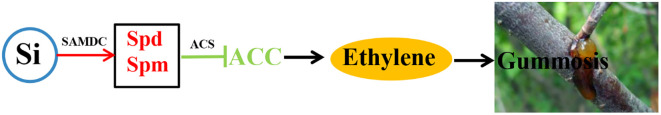
Silicon-mediated polyamine inhibition of ethylene synthesis to inhibit gummosis. Target induction is depicted by solid arrows, whereas inhibition is depicted by blocked lines. Red indicates upregulation, and green indicates downregulation. (Si: silicon; SAMDC: S-adenosine methionine decarboxylase; ACS: ACC synthase; Spd: spermidine; Spm: spermine).

In conclusion, ethylene is involved in peach gummosis; it enhances the enzymatic activity of cell wall-degrading and promotes gummosis. Silicon upregulated the expression of *PpSAMDC*, promoted the accumulation of Spd and Spm. Simultaneously, polyamine reduced the expression of *PpACS1* and the enzyme activity of ACS, then reduced the ethylene synthesis. (**Figure 6**). So silicon restricted gummosis development by activating ployamines biosynthesis and inhibiting ethylene synthesis in peach. However, the specific molecular mechanism of silicon regulating the synthesis of polyamines and ethylene to inhibit peach gums needs further study. For example, it is unclear how silicon regulates the expression and synthesis of polyamine and ethylene synthesis genes and the specific mechanism of polyamine and ethylene inhibiting gumming.

## Data availability statement

The data presented in the study are deposited in online repositories. The data presented in the study are deposited in the NCBI repository, accession number PRJNA899649.

## Author contributions

HG: Data curation, writing-original draft. XW: Conceptualization, data curation. XY: Investigation, methodology. MS: Conceptualization, investigation. JL: Investigation. FP: Conceptualization, methodology, project administration, supervision, writing-review, editing. YX: Conceptualization, methodology, supervision, writing – review, editing. All authors contributed to the article and approved the submitted version.

## Funding

This work was supported by the National Key Research and Development Program of China (2020YFD1000203) and the earmarked fund for China Agriculture Research System (No. CARS-30-2-02)

## Conflict of interest

The authors declare that the research was conducted in the absence of any commercial or financial relationships that could be construed as a potential conflict of interest.

## Publisher’s note

All claims expressed in this article are solely those of the authors and do not necessarily represent those of their affiliated organizations, or those of the publisher, the editors and the reviewers. Any product that may be evaluated in this article, or claim that may be made by its manufacturer, is not guaranteed or endorsed by the publisher.
