# Supplementary material for: Silicon inhibits gummosis by promoting polyamine synthesis and repressing ethylene biosynthesis in peach
Source: Front Plant Sci. 2022 Nov 28;13:986688. doi: 10.3389/fpls.2022.986688 (PMC9744191; doi:10.3389/fpls.2022.986688)
Supplement: Supplementary file 1 [file DataSheet_1.docx]

**
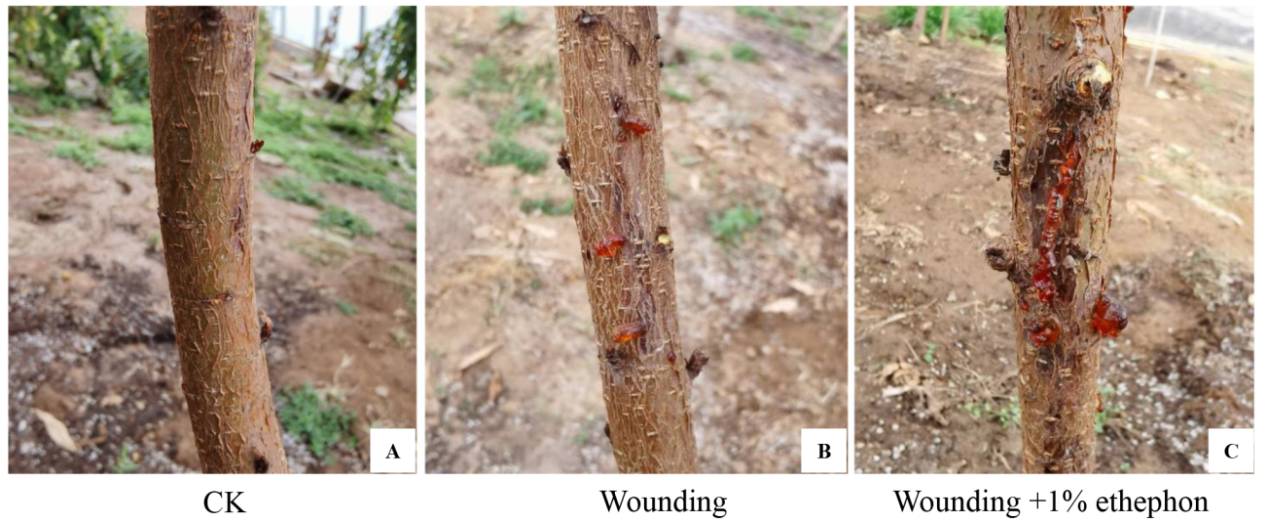
**

Fig S1. Gum formation induced by wounding and ethephon (1%, w/w) ten days after treatment. (A) Control. (B) Wounding. (C) Wounding +1% ethephon.

**
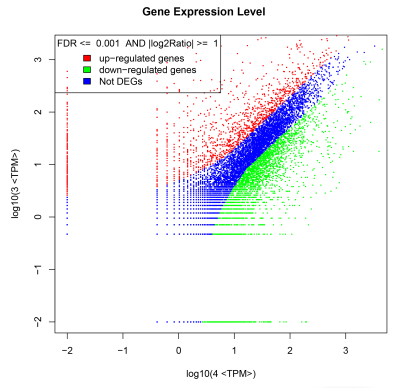

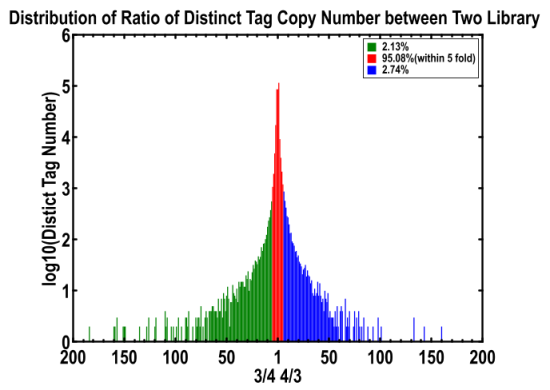

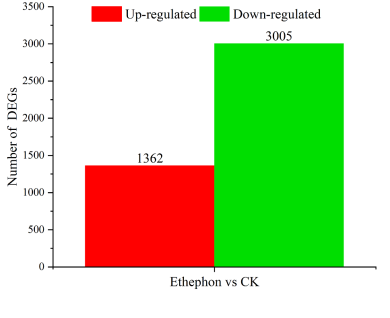
**

B

C

A

Fig S2. Tag and gene expression levels of CK and ethephon treatment groups. (A) Gene expression levels in CK and ethephon treatment groups (fold-change≥2, FDR< 0.1). (B) Distribution of tag expression ratios between CK and ethephon-treated samples. (C) Number of up- or down-regulated genes (red is upregulated; green is downregulated).


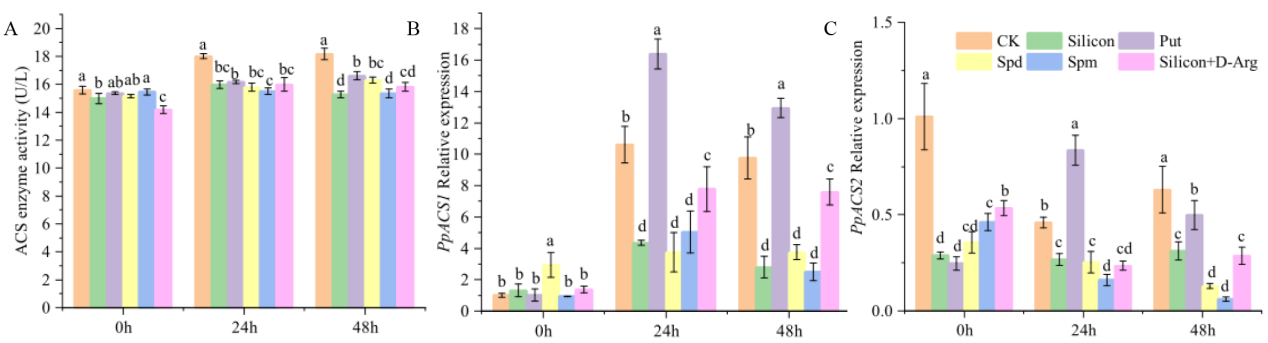


Fig S3. Spd and Spm inhibit ACS enzyme actuvity in peach shoots. Enzyme activity of ACS (A) in peach shoots with different treatments; Relative expression of PpACS1 (B) and PpACS2 (C) in peach shoots under different treatments. The peach shoots treated with water, Na_2_SiO_3_ (0.6 mmol/L), Put (0.1 mmol/L), Spd (0.1 mmol/L), Spm (0.1 mmol/L) or Na_2_SiO_3_ +D-Arg (10 mg/L).


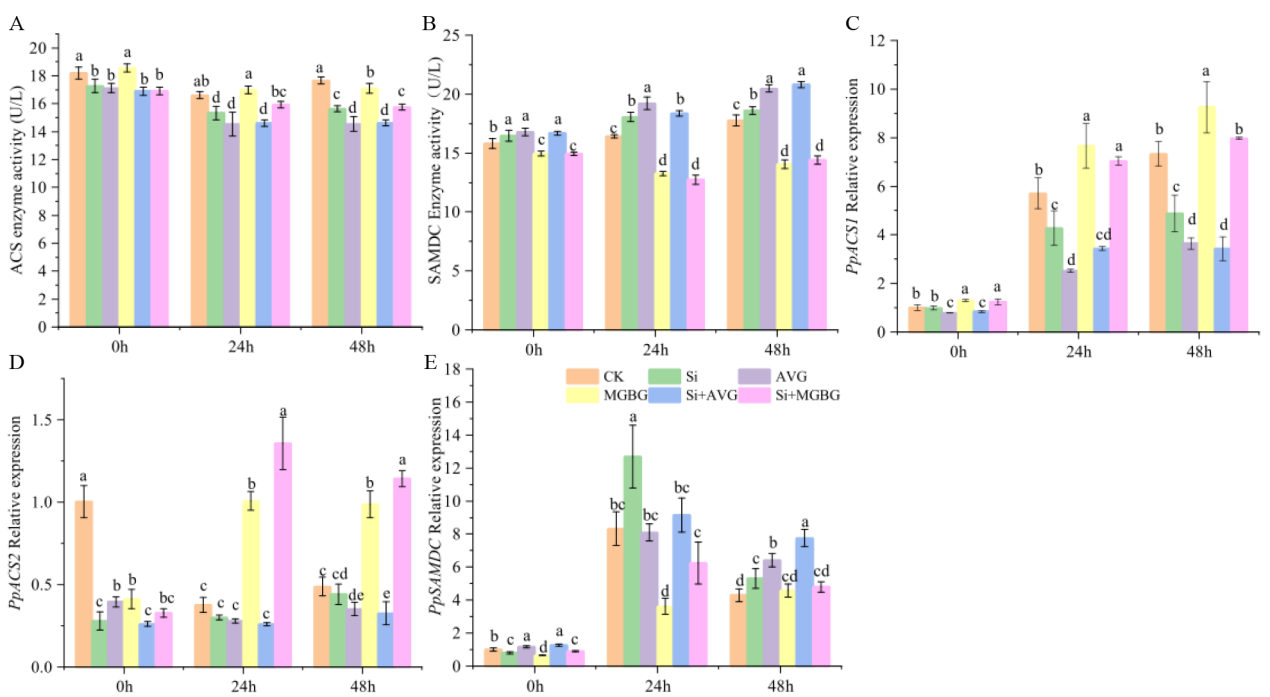


Fig S4. Silicon regulates the expression of ethylene and polyamine synthesis genes. Enzyme activity of ACS (A) and SAMDC (B) in peach shoots under different treatments; Relative expression of *PpACS1* (C), *PpACS2* (D) and *PpSAMDC* (E) in peach shoots under different treatments. The peach shoots were treated with water, Na_2_SiO_3_ (0.6 mmol/L), AVG (0.5 mmol/L) and MGBG (0.5 mmol/L) individually or in combination, and samples were taken for analysis at 0h, 24h, and 48h after treatment.
